# Variability in vancomycin levels in an intensive care population explained by variability in clearance

**DOI:** 10.1186/2197-425X-3-S1-A629

**Published:** 2015-10-01

**Authors:** PJ Lans, Y Lao, H Sporsem, E von der Lippe, V Bakke, K Horvig, H Nyrerød, J Bugge, E Helset

**Affiliations:** Department of Anesthesiology, Oslo University Hospital, Oslo, Norway; Oslo Hospital Pharmacy, Oslo, Norway; Department of Infectious Diseases, Oslo University Hospital, Oslo, Norway; Medical School, Oslo University Hospital, Oslo, Norway

## Introduction

Vancomycin levels are frequently subtherapeutic or too high in critically ill patents.

## Objectives

To study how variability in clearance: creatinine clearance (CrCL), total vancomycin clearance (totVCL) and extracorporal clearance (CRRTCL) correlated with vancomycin through levels.

## Methods

In a prospective observational study we included patients started on vancomycin, from four different intensive care units (ICU) at Oslo University Hospital. Blood samples as well as ultrafiltrate samples from patients on continuous renal replacement therapy (CRRT), were collected in one dosage interval on three consecutive days 24 hours(hrs) from start. The observation time was 72 hrs. Vancomycin concentrations were determined using a commercial assay (Cobas C Systems Roche). CrCL, totVCL and CRRTCL were calculated for each dosage interval.

## Results

76 patients were included, 48 non-CRRT and 28 CRRT: 15 on continuous venovenous hemodialysis (CVVHD) and 13 on continuous venovenous hemofiltration (CVVH). In the CRRT group the effluent flow rate was 2099 ± 435 ml/hr (SD). Age mean was 50.8 ± 15.1 years, 74 % men and 26 % women. SAPS mean was 47.2 ± 16.0. The reasons for admittance to the ICU were trauma 25%, postoperative complications 42%, septicaemia 15% and respiratory failure 18%. The average vancomycindose at the points of measurements was 1207,4 ± 550,4 mg. In the non-CRRT group the mean CrCL was 117 ± 9 ml/min(SEM), and the totVCL was 88 ± 6 ml/min. There were no significant differences between the measurements at 24,48 and 72 hrs. A significant correlation was found between CrCL and totVCL in the non-CRRT group as expected. In the CRRT group the mean totVCL was 51 ± 2 ml/min, a significant reduction in clearance with 43% as compared to the non-CRRT group. The mean CRRTCL was 26 ± 3 ml/min. No significant differences was observed between the sieving coefficients in CVVH (0.71 ± 0.001) compared to CVVHD (0.69 ± 0.02). There was a significant negative correlation between totVCL and vancomycin through concentrations in both the non-CRRT and the CRRT group, but a stronger negative correlation in the non-CRRT group (p < 0.001).

## Conclusions

In the CRRT group, clearance of vancomycin by CRRT contributed to 50% of totVCL independent of CRRT mode. The reduced totVCL in patients on CRRT, should lead to reduced maintenance dose of vancomycin to avoid to high through concentrations. Measurement of CrCL may be useful in non-CRRT critically ill patients to avoid subtherapeutic vancomycin levels.

